# Defective Mitochondrial Dynamics Is an Early Event in Skeletal Muscle of an Amyotrophic Lateral Sclerosis Mouse Model

**DOI:** 10.1371/journal.pone.0082112

**Published:** 2013-12-06

**Authors:** Guo Luo, Jianxun Yi, Changling Ma, Yajuan Xiao, Frank Yi, Tian Yu, Jingsong Zhou

**Affiliations:** 1 Department of Molecular Biophysics and Physiology, Rush University School of Medicine, Chicago, Illinois, United States of America; 2 Zunyi Medical College, Zunyi, China; National Institute of Health, United States of America

## Abstract

Mitochondria are dynamic organelles that constantly undergo fusion and fission to maintain their normal functionality. Impairment of mitochondrial dynamics is implicated in various neurodegenerative disorders. Amyotrophic lateral sclerosis (ALS) is an adult-onset neuromuscular degenerative disorder characterized by motor neuron death and muscle atrophy. ALS onset and progression clearly involve motor neuron degeneration but accumulating evidence suggests primary muscle pathology may also be involved. Here, we examined mitochondrial dynamics in live skeletal muscle of an ALS mouse model (G93A) harboring a superoxide dismutase mutation (SOD1^G93A^). Using confocal microscopy combined with overexpression of mitochondria-targeted photoactivatable fluorescent proteins, we discovered abnormal mitochondrial dynamics in skeletal muscle of young G93A mice before disease onset. We further demonstrated that similar abnormalities in mitochondrial dynamics were induced by overexpression of mutant SOD1^G93A^ in skeletal muscle of normal mice, indicating the SOD1 mutation drives ALS-like muscle pathology in the absence of motor neuron degeneration. Mutant SOD1^G93A^ forms aggregates inside muscle mitochondria and leads to fragmentation of the mitochondrial network as well as mitochondrial depolarization. Partial depolarization of mitochondrial membrane potential in normal muscle by carbonyl cyanide p-trifluoromethoxyphenylhydrazone (FCCP) caused abnormalities in mitochondrial dynamics similar to that in the SOD1^G93A^ model muscle. A specific mitochondrial fission inhibitor (Mdivi-1) reversed the SOD1^G93A^ action on mitochondrial dynamics, indicating SOD1^G93A^ likely promotes mitochondrial fission process. Our results suggest that accumulation of mutant SOD1^G93A^ inside mitochondria, depolarization of mitochondrial membrane potential and abnormal mitochondrial dynamics are causally linked and cause intrinsic muscle pathology, which occurs early in the course of ALS and may actively promote ALS progression.

## Introduction

ALS is an adult onset and fatal neuromuscular disease characterized by the progressive loss of motor neuron (MN) and skeletal muscle atrophy. Most cases of ALS are sporadic (SALS), with about 10% being familial (FALS) [1]. Both SALS and FALS manifest similar pathological and clinical phenotypes, suggesting that different initiating molecular insults promote a similar neurodegenerative process. Many cases of FALS (20-25%) are associated with mutations in the Cu/Zn-superoxide dismutase gene (SOD1) [1]. Transgenic mice harboring human ALS-causing SOD1 mutations recapitulate the neuronal and muscle impairment of human ALS patients and thus these mice are widely used by the ALS research community [2].

The common pathological hallmark of ALS is the death of motor neuron. However, defects present in other cell types may also actively contribute to the disease progression [3]. Motor neurons communicate with skeletal muscle at the site of neuromuscular junction (NMJ). Retrograde signaling from muscle-to-neuron is critical for axonal growth and maintenance of NMJ [4,5]. ALS has been described as a “distal axonopathy”, which affects the axon and NMJ in ALS transgenic mouse model at the age prior to significant loss of neuronal bodies and the onset of muscle atrophy [6,7]. It is possible that an intrinsic muscle defect early in the course of ALS promotes or contributes to the motor axonal withdrawal. While many ALS studies focus on neurodegeneration, just a few have explored the possible contribution of primary muscle defects. The gene expression profile of ALS muscle is significantly different from that of the muscle with axotomy-induced denervation [8], suggesting there are ALS muscle defects that are independent of axonal withdrawal. Two research groups independently produced transgenic mouse models with muscle-restricted expression of ALS-causing mutant proteins (SOD1^G93A^). Both mouse models showed muscle degeneration [9,10], but only one had motor neuron degeneration [10]. Interestingly, one of those research groups showed that muscle-restricted expression of wild type SOD1 also induced motor neuron degeneration [10]. This result is contradictory to that of an early study, in which the transgenic mice with systematic overexpression of wild type SOD1 do not develop overt ALS symptoms [2]. In addition, study from Miller et al showed that partial reduction of the expression of mutant SOD1 in muscle did not affect the disease onset or survival in ALS transgenic mice [11]. Therefore, the role of skeletal muscle defects in ALS onset and progression is still poorly understood. 

The high energy demand of muscle contraction is met by a large endowment of mitochondria that occupy 10–15% of muscle fiber volume [12]. Morphological and biochemical analyses reveal the existence of defective mitochondria in skeletal muscle of ALS patients (reviewed in [13]). Since all patients tested were at symptomatic stages, it is not clear whether these mitochondrial defects were the cause or consequence of ALS muscle atrophy[14]. Biochemical studies on skeletal muscle derived from ALS transgenic mice also report altered mitochondrial respiratory properties [15–18]. We previously conducted functional studies on live muscle fibers of ALS transgenic mice carrying mutant SOD1^G93A^ (G93A) and found that a portion of muscle fibers had depolarized mitochondria near NMJ [19,20]. This NMJ localized mitochondrial depolarization could be caused by a primary defect or localized axonal withdrawal. Here, we examine mitochondria in G93A model muscle in more detail and determine if ALS-like muscle defects can occur independent of the axonal withdrawal. 

Mitochondria are morphologically highly dynamic organelles that are constantly remolded by fusion and fission processes [21]. This phenomenon, known as mitochondrial dynamics, defines normal mitochondrial morphology and distribution, cell bioenergetics and cell death [22]. Abnormal mitochondrial dynamics is implicated in various neurodegenerative disorders [23,24]. Recent studies show that abnormal mitochondrial dynamics contribute to the degeneration of cultured motor neurons overexpressing ALS-causing mutant SOD1 [25,26]. Because of the strict structural arrangement of skeletal muscle, the existence and relevance of mitochondrial movement is less understood in skeletal muscle [27]. Thus, the role of mitochondrial dynamics in normal muscle physiology is still unclear as is its role during ALS progression. Here, we investigate mitochondrial dynamics in ALS skeletal muscle for the first time. Using photoactivatable and mitochondria-targeted fluorescent proteins, we report that the skeletal muscle of an ALS mouse model (G93A) has abnormal mitochondrial dynamics before the onset of overt disease (a stage without significant motor axonal withdrawal, [2]). We also show that overexpression of muant SOD1^G93A^ alters mitochondrial dynamics in skeletal muscle of normal mice, where there is no motor neuron degeneration. Remarkably, only the mutant SOD1^G93A^ (not the wild type SOD1) overexpression results in formation of protein aggregates inside mitochondria, mitochondrial fragmentation and abnormal dynamics. The abnormal mitochondrial dynamics evoked by SOD1^G93A^ overexpression is associated with depolarization of mitochondrial membrane potential and augmented mitochondrial fission process. Overall, our data indicate the SOD1^G93A^ mutation directly causes abnormal mitochondrial dynamics in skeletal muscle even in the absence of motor neuron degeneration. Thus, abnormal mitochondrial dynamics in muscle may contribute to SOD1 mutation-associated ALS disease onset and progression. 

## Experimental Procedures

### Animals used and gene transfection in skeletal muscle of adult mice

G93A [2] and age matched normal mice were used in this study. The electroporation protocol was modified from [28] and [19]. The anaesthetized mice were injected with 10 µl of 2 mg/ml hyaluronidase dissolved in sterile saline at the ventral side of hind paws through a 29-gauge needle. One hour later, 5-10 µg plasmid DNA in 10 µl sterile saline were injected into the same sites. 15 min later, two electrodes (gold plated stainless steel acupuncture needles) were placed at the starting line of paw and toes, separated ~ 9 mm. 20 pulses of 100 V/cm at 20 ms/pulse were applied at 1 Hz (ECM 830 Electro Square Porator, BTX). 7 days later, the animal was euthanized by CO_2_ inhalation, and the flexor digitorum brevis (FDB) muscles were removed for functional studies. All experiments were carried out in strict accordance with the recommendation in the Guide for the Care and Use of Laboratory Animals of the National Institutes of Health. The protocol was approved by the IACUC of Rush University. The injection and electroporation procedures were performed under the anesthesia using constant isoflurane inhalation. All efforts were made to minimize suffering.

### Muscle fiber preparation

Individual muscle fibers were isolated following the protocol used previously [19,20]. Briefly, FDB muscles were digested in modified Krebs solution (0 Ca^2+^) plus 0.2% type I collagenase for 55 min at 37°C. Following collagenase treatment, muscle fibers were stored in an enzyme-free Krebs solution at 4°C, and used for imaging studies within 24 hours. 

### Plasmid construction

The cDNAs of SOD1 and SOD1^G93A^ were amplified from pBluescript-SOD1 and pBluescript-SOD1^G93A^ (gifts from Dr. Han-Xiang Deng, Northwestern University). To construct SOD1-GFP and SOD1^G93A^-GFP, the amplified SOD1 and SOD1^G93A^ were subcloned into pEGFP-N1 (Clontech) at sites of BamH I and Nhe I. To make mt-SOD1-Dendra and mt-SOD1^G93A^-Dendra, the cDNAs of SOD1 or SOD1^G93A^ was inserted into pDendra2-N (Evrogen) at sites of BamH I and Hind III. The mitochondrial targeting sequence consisting of 29 amino acids amplified from pCMV/myc/mito (invitrogen) was subsequently fused at the 5’ end of SOD1-Dendra and SOD1^G93A^-Dendra constructs. All final constructs were confirmed by sequencing. mt-PAGFP was purchased from Addgene (#23348).

### Fluorescence dye loading and confocal microscopic imaging

FDB muscle fibers were incubated with 50 nM tetramethylrhodamine ethyl ester (TMRE) for 10 min at 25 °C for visualization of mitochondrial membrane potential. Photoactivation with 364 nm laser light was used to activate PAGFP to show GFP green fluorescence. GFP and TMRE images were simultaneously recorded. GFP was excited at 488 nm and its emitted fluorescence was collected at 490-540. TMRE was excited at 543 and its emitted fluorescence was collected at 560-620. Before photoactivation Dendra is a green fluorescent protein. Photoactivation with 364 nm laser light converts Dendra from a green to a red fluorescent protein. Thus after photoactivation Dendra was excited at 543 and its emitted fluorescence was collected at 560-620. A confocal microscope (SP2-AOBS Leica Microsystem, Gemany with a 63X, 1.2 NA water-immersion objective) capable of line-interleaving images excited with different lasers, was used. TMRE was purchased from Invitrogen.

### Application of pharmacological reagents

The mitochondrial fission inhibitor, Mdivi-1 [29] was applied by intraperitoneal injection in the dose of 50 mg/kg/day for 7 days before FDB muscles were removed for analysis of mitochondrial dynamics. Mdivi-1 was dissolved in di-methyl sulfoxide (DMSO) at the concentration of 12.5%. Then 8 µl Mdivi-1 (1 mg) solution was dissolved in 200 µl saline for intraperitoneal injection. The same dilution of DMSO in normal saline was applied as vehicle control. In order to analyze the effect of mitochondrial membrane potential on mitochondrial dynamics, a proton ionophor (FCCP, 200 nM) was applied to muscle fibers in culture dishes to partially depolarize mitochondrial inner membrane potential. Chemicals were purchased from Sigma. 

### Immunoblot assay

Tibialis anterior muscles from normal and G93A mice were collected and homogenized in protein extraction buffer containing protease inhibitor cocktail (Thermo Scientific) using motorized homogenizer (Wheaton). Protein concentrations were determined by BCA protein assay (Thermo Scientific). Protein samples (~30 µg) were subject to 8% SDS-polyacrylamide gel electrophoresis. The protein was transferred into an Immobilon PVDF membrane (Millipore, Bedford, MA). The primary antibodies used to probe the protein samples are anti-Mfn1 (1:2500) and anti-Mfn2 (1:5000) from Abcam; anti-Drp1 (1:5000) from Cell Signaling, and anti-tubulin (1:10000) from Santa Cruz Biotechnology. The C-SOD1 antibody was a generous gift from Dr. Han-Xiang Deng [30]. Results were visualized with ECL reagents (Thermo). Densitometry evaluation was conducted using Image/J software (NIH).

### Image processing and data analysis

IDL 7.0 (IDL, ITT Visual Information Solutions) was used for image processing. Sigmaplot 11.0 and Microsoft Excel were used for data analysis. Data are represented as mean ± S.E.M. Statistical significance was determined by Student’s t test.

## Results

### Abnormal mitochondrial dynamics in skeletal muscle of G93A mice before disease onset

**Figure 1:**
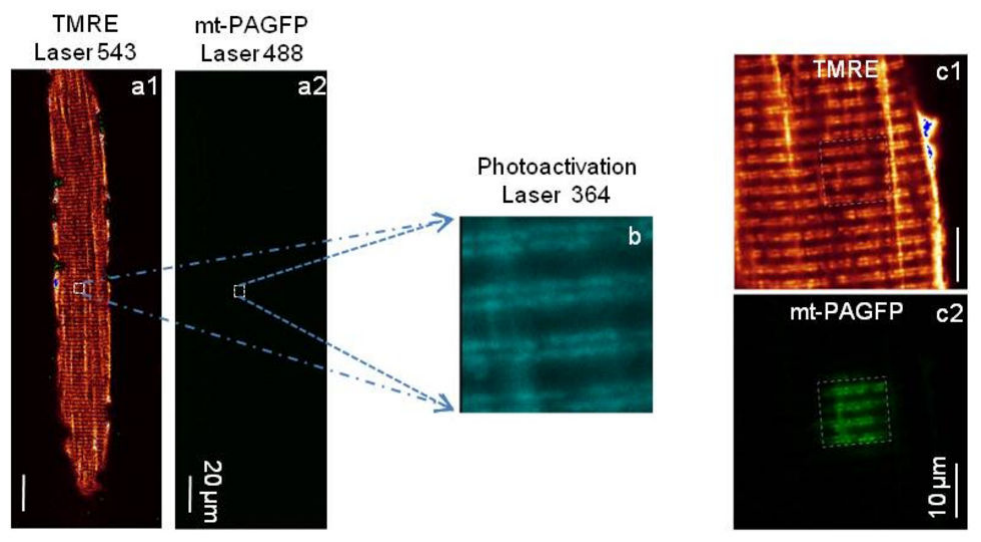
Photoactivation of mt-PAGFP. Simultaneous recording of TMRE (a1) and mt-PAGFP (a2) images. Before photoactivation, there is no GFP fluorescence of mt-PAGFP detected in **a2**. A small area (~ 10 X 10 µm^2^) of the fiber was photoactivated by a 364 nm laser (**b**). After photoactivation, the fiber was imaged again simultaneously for both TMRE (c1) and mt-PAGFP (c2) at the fiber region including the area with photoactivated GFP. Note the visible GFP fluorescence of mt-PAGFP in **c2** recorded at the same laser intensity as in **a2**.

The ALS mouse model (G93A) is asymptomatic until after 3 months of age [2]. To examine mitochondrial dynamics prior to ALS onset, experiments here were done on two-month old G93A and age-matched normal mice (control). A mitochondria-targeted photoactivatable green fluorescent protein (mt-PAGFP) was transfected into the FDB muscle of both G93A and control mice in order to monitor mitochondrial dynamics in skeletal muscle. Seven days post-transfection, the transfected FDB muscles were collected and enzyme-digested to isolate individual muscle fibers for confocal imaging studies. The isolated live muscle fibers were incubated with TMRE to visualize mitochondria (Figure **1, a1**). TMRE and mt-PAGFP were then excited at 543 nm and 488 nm respectively. Interleaved excitation and spectrally separated emission wavelengths permitted simultaneous recording of the TMRE and mt-PAGFP signals. Before photoactivation, mt-PAGFP showed no GFP fluorescence (Figure **1, a2**). Photoactivation of a small region (~10 µm x 10 µm) of the muscle fiber by a 364 nm laser light (Figure **1, b**) evoked mt-PAGFP fluorescence in this region (Figure **1, c2**). Comparison of TMRE and mt-PAGFP signals demonstrates that mt-PAGFP is indeed expressed inside mitochondria (Figure **1, c1 and c2**). 

**Figure 2:**
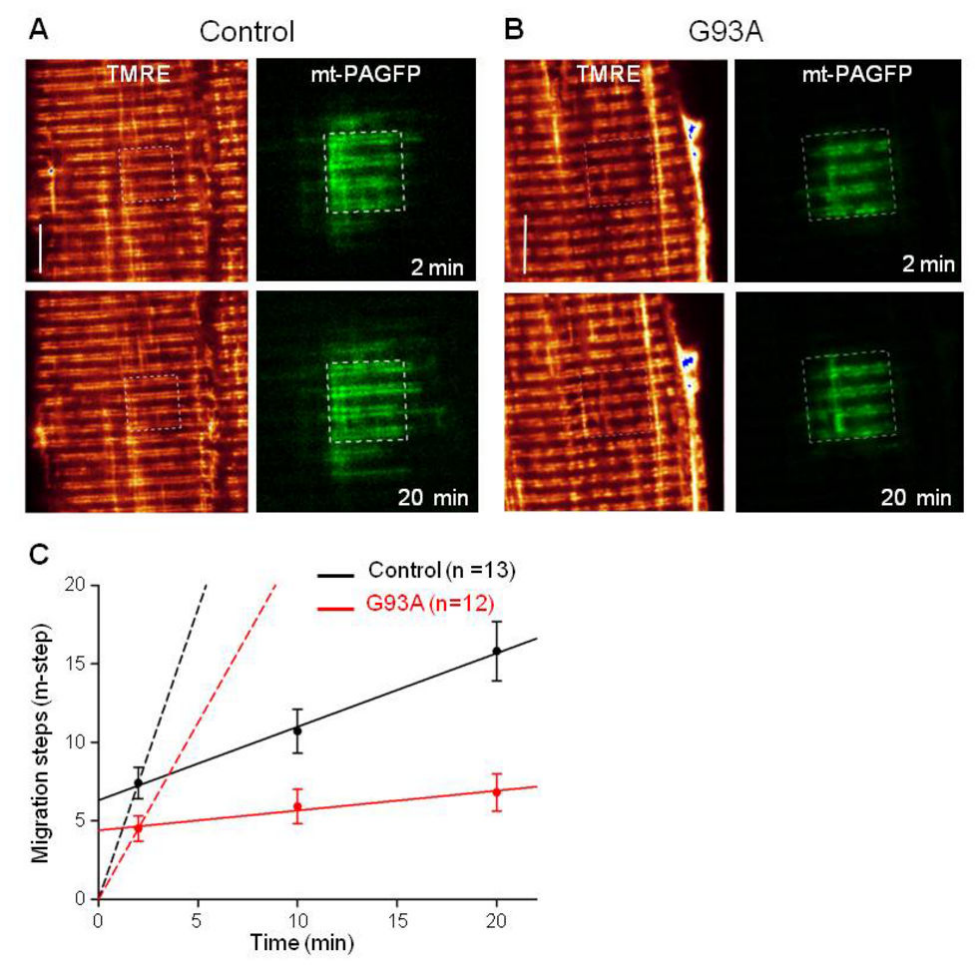
Migration of mt-PAGFP out of the photoactivation region of muscle fibers. Representative images of normal (control) (**A**) and G93A muscle fibers (**B**). The first image was taken 2 min after the photoactivation because it took a couple of minutes to reconfigure the confocal microscope system to conduct the simultaneous scan. The photoactivation region inside the fiber was marked with the box (white dashed line). Bar: 10 µm. (**C**) is the time course of mt-PAGFP migration. The linear regression was used to fit the data to evaluate the migration rate. Solid lines are the linear regression of the data after 2 minutes of the photoactivation. By assuming the migration step is zero at time zero, the linear regression was applied to determine the migration rate during the first 2 min (dashed line). The migration rates are listed in Table **1**. (P<0.01).

Mitochondrial dynamics were evaluated by examining the time-dependent migration of mt-PAGFP out of the original photoactivation area. Representative control and G93A images are shown in Figure **2 A and B**. Migration of mt-PAGFP was evaluated in both longitudinal and transversal directions in muscle fibers. The length of one migration step (m-step) was defined as the average sarcomere length (2.2 µm). The first image was taken 2 min after the photoactivation because of the time needed to reconfigure the microscope to simultaneously record the TMRE and GFP images. The mt-PAGFP in control muscle fibers moved 8.4 ± 1.1 m-steps in 18 min (n=13), it only moved 2.3 ± 0.4 m-steps in G93A muscle fibers (n=12, P<0.01). Linear regression fitting was used to define the rate of migration (solid line in Figure **2C**). As listed in Table **1**, the migration rates were 0.47 m-steps/min (control) and 0.13 m-steps/min (G93A) (P<0.01) during the 18-min migration. The initial values of migration steps measured in the first images (at 2 min) are also significantly different between G93A and the control (Figure **2C**). By assuming the migration is zero at time zero, we also made linear regression fitting and defined the migration rate in the first 2 minutes (dashed lines in Figure **2C**). The migration rates in the first 2 minutes were 3.7 m-steps/min (control) and 2.25 m-steps/min (G93A). Thus, mt-PAGFP migration in G93A muscle was 1.6-fold slower in the first 2 minutes and 3.6-fold slower later on (Table **1**). The migration rates in the first 2 min and after the first 2 min represent different features of mitochondrial dynamics in skeletal muscle. The detail is discussed in the DISCUSSION section. 

**Table 1:** Migration rates of fluorescent proteins after the photoactivation.

**Conditions**	**Migration rate (m-step/min)**	
	**during first 2 min**	**after 2 min**
mt-PAGFP in normal muscle	3.70	0.47
mt-PAGFP in G93A muscle	2.25	0.13
**Ratio of migration rates: normal/G93A**	**1.6**	**3.6**
mt-SOD1-Dendra overexpressed in normal muscle	8.10	0.46
mt-SOD1^G93A^-Dendra overexpressed in normal muscle	5.05	0.19
**Ratio of migration rates: SOD1/SOD1^G93A^**	**1.6**	**2.4**
mt-SOD1-Dendra overexpressed in normal muscle (FCCP)	6.25	0.25
**Ratio of migration rates: SOD1/SOD1 in FCCP**	**1.3**	**1.8**
mt-SOD1^G93A^-Dendra overexpressed in normal muscle (Mdivi-1)	7.75	0.55
mt-SOD1^G93A^-Dendra overexpressed in normal muscle (DMSO)	4.35	0.24
**Ratio of migration rates: SOD1^G93A^(Mdivi-1)/SOD1^G93A^ (DMSO)**	**1.8**	**2.3**

### Mutant SOD1^G93A^ protein causes abnormal mitochondrial dynamics in skeletal muscle

The reduced mitochondrial dynamics observed in skeletal muscle of G93A mice could be a consequence of the motor neuron withdrawal from muscle. However, two-month old G93A mice are asymptomatic and have no significant motor axonal withdrawal [2]. Thus, we hypothesize that expression of mutant SOD1^G93A^ in skeletal muscle directly (i.e. neuron independent) alters mitochondrial dynamics in skeletal muscle of G93A mice before ALS onset. 

**Figure 3:**
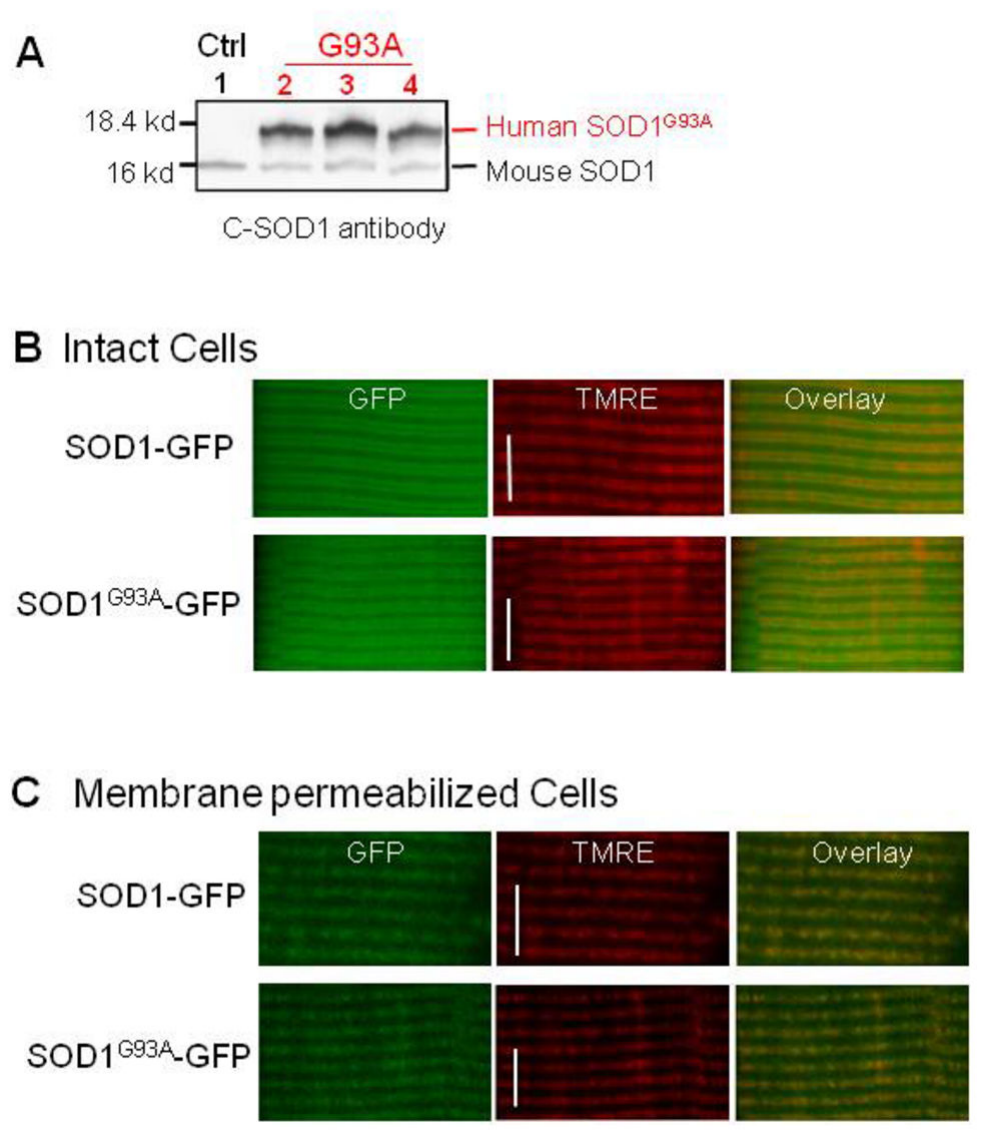
Cytosolic SOD1 proteins can be transported into mitochondria of skeletal muscle. (**A**) Western blot to show the expression of human SOD1^G93A^ in the skeletal muscle of G93A mice. C-SOD1 antibody recognizes both mouse and human SOD1. Lane 1: Control muscle; Lane 2-4: G93A muscle (2,3 and 4-month old). Note the skeletal muscle of G93A mice expresses human SOD1^G93A^ at all ages. (**B**) Intact normal muscle fibers expressing SOD1-GFP or SOD1^G93A^-GFP. Note, in addition to the cytosolic expression, both SOD1-GFP and SOD1^G93A^-GFP show mitochondrial targeting. (**C**) After cell membrane permeabilization, the cytosolic GFP was lost. The overlay of TMRE and remaining GFP signals indicate the expression of SOD1-GFP or SOD1^G93A^-GFP inside mitochondria. Bar: 10 µm.

We examined the expression of mutant SOD1^G93A^ in skeletal muscle of the G93A mouse model by immunoblot assay. The C-SOD1 antibody used here recognizes both human and mouse SOD1. Since human SOD1 is larger than mouse SOD1, these two proteins are easily differentiated in the blot [30]. As demonstrated in Figure **3A** (Lane 2-4), the G93A muscle expresses human SOD1^G93A^ at all disease stages. The muscle from a control normal mouse (Lane 1) had no human SOD1^G93A^ expression. We then determined whether mutant SOD1^G93A^ is associated with mitochondria in skeletal muscle. SOD1 is a cytosolic protein that can be transported into mitochondria in some cell types [31]. Immunohistochemical analysis of motor neurons in G93A mice showed that mutant SOD1 accumulates inside swollen mitochondria [30]. In an earlier study, we reported the existence of swollen mitochondria in G93A muscle [19], but did not evaluate SOD1 localization. To trace SOD1 targeting in live muscle fibers, we constructed two plasmids for SOD1-GFP and SOD1^G93A^-GFP fusion proteins. Both plasmids were transfected into the FDB muscle of normal mice respectively. Seven days after transfection, muscles were collected and single muscle fibers were isolated. Fibers expressing SOD1-GFP or SOD1^G93A^-GFP were incubated with TMRE to visualize mitochondria. Overlay of TMRE and SOD1-GFP or SOD1^G93A^-GFP images (Figure **3B**) show that, in addition to cytosolic expression, both SOD1-GFP and SOD1^G93A^-GFP proteins are targeted to mitochondria. The mitochondrial targeting of SOD1-GFP and SOD1^G93A^-GFP was further confirmed by permeabilizing the cell membrane. After permeabilization, the cytosolic fluorescent protein was lost and the fluorescent protein inside mitochondrial remained. Consequently, the intracellular GFP fluorescence was reduced by 90%. Increased laser intensity was required to demonstrate that the remaining GFP signal was from mitochondria (Figure **3C**). No mitochondrial auto-fluorescence was observed in non-transfected fibers at the same laser intensity and digital gain. These data indicate that both wild type SOD1 and mutant SOD1^G93A^ are indeed transported into mitochondria in skeletal muscle. 

**Figure 4:**
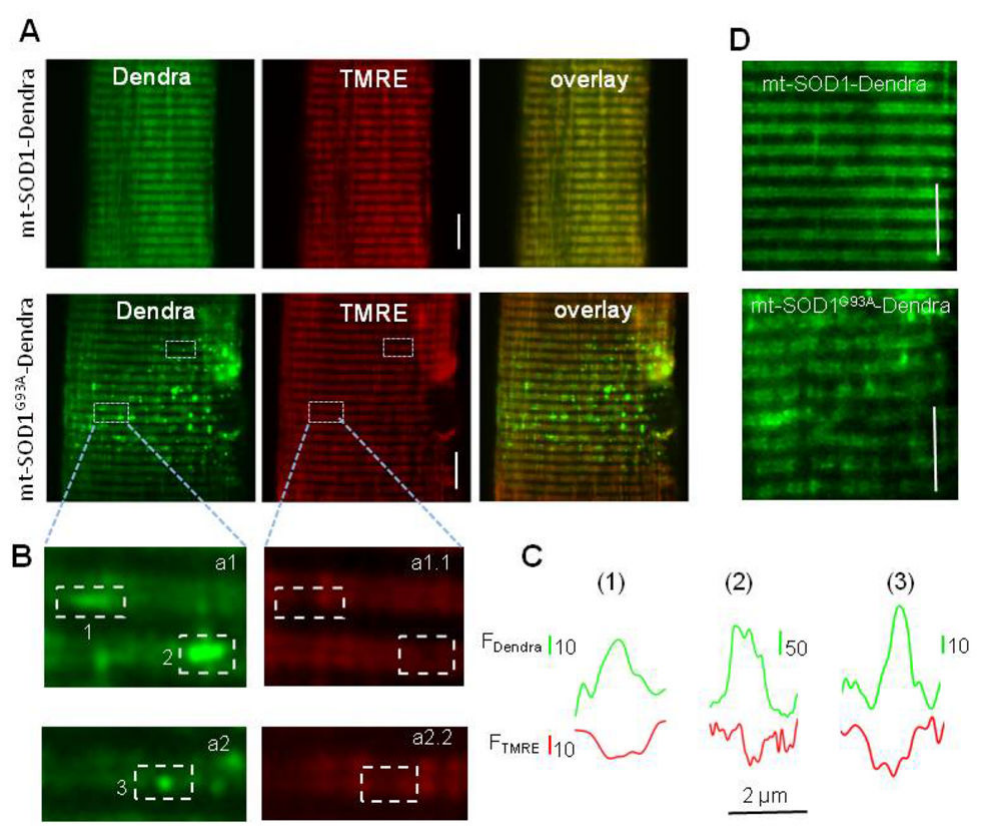
Expression of mitochondria-targeted SOD1 in skeletal muscle of normal mice. (**A**) Normal muscle fibers transfected with mt-SOD1-Dendra or mt-SOD1^G93A^-Dendra. Both proteins preferentially target to mitochondria. 90% of fibers expressing mt-SOD1^G93A^-Dendra show aggregated fluorescent proteins inside mitochondria (n=73), while not a single fiber expressing wild type mt-SOD1-Dendra has protein aggregates (n=36). (**B**) Enlarged boxes (**a1** and **a2**) taken from the mt-SOD1^G93A^-Dendra fiber show that fiber regions (1, 2 and 3) with fluorescent protein aggregates have reduced TMRE staining (**a1.1** and **a2.2**). (**C**) Fluorescence profiles of Dendra (**F_Dendra_**, green) and TMRE (**F_TMRE_**, red) at those three regions indicate that depolarized mitochondria are at the site of protein aggregates. (**D**). Representative images of normal muscle fibers expressing mt-SOD1-Dendra or mt-SOD1^G93A^-Dendra. 63% of mt-SOD1^G93A^-Dendra fibers show fragmented mitochondria comparing to only 11% of mt-SOD1-Dendra fibers. Bar: 10 µm.

**Figure 5:**
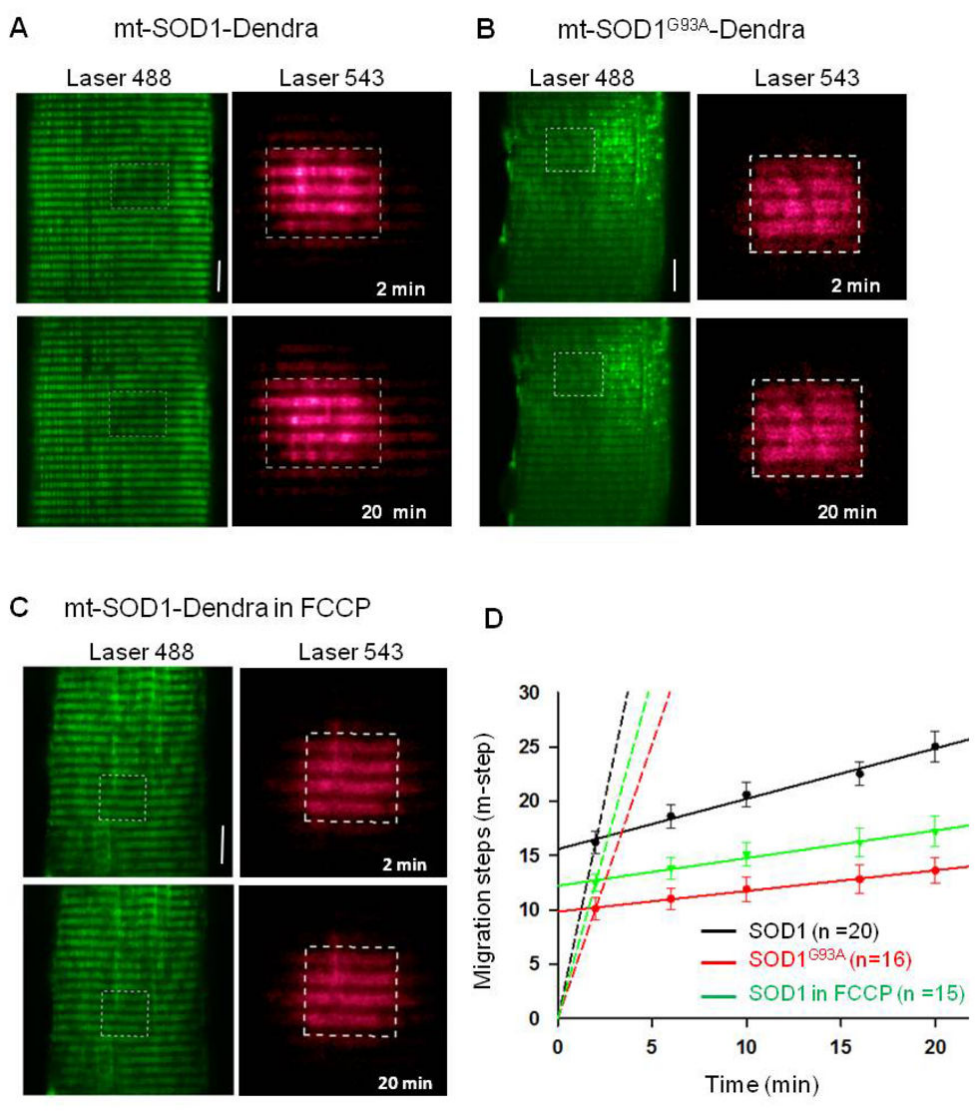
Migration of mt-SOD1-Dendra and mt-SOD1^G93A^-Dendra in normal muscle fibers. Representative images of muscle fibers expressing mt-SOD1-Dendra (**A**) or mt-SOD1^G93A^-Dendra (**B**). Before photoactivation Dendra was a green fluorescent protein (green images in left panels). A small region of the fiber was photoactivated. The photoactivation region in the fiber was marked with the box (white dashed line). After the photoactivation Dendra was converted to a red fluorescent protein (red images in right planes). (**C**) Migration of mt-SOD1-Dendra in the presence of 200 nM FCCP. Bar: 10 µm. (**D**) The migration time course of the red fluorescent protein. Solid lines are the linear regression of the data after 2 minutes of the photoactivation. Dashed lines are the linear regression of the data in the first 2 minutes, assuming the migration step is zero at time zero. The migration rates are listed in Table **1**. (P<0.01).

Next, we investigated whether mutant SOD1^G93A^ inside muscle mitochondria directly induces abnormal mitochondrial dynamics in the absence of motor neuron degeneration by overexpressing SOD1^G93A^ in the skeletal muscle of normal mice. Since only a small portion of cytosolic-expressed SOD1-GFP or SOD1^G93A^-GFP ends up inside mitochondria after 7 days of transfection (Figure **3C**), the toxicity of the mutant protein on mitochondria may not be detectable at this time. In order to better evaluate the toxic effect of SOD1^G93A^ on mitochondrial structure and function, we targeted the mutant protein specifically to mitochondria by constructing a plasmid, mt-SOD1^G93A^-Dendra. In this plasmid, a mitochondrial targeting peptide was attached to the N-terminus of SOD1^G93A^ and a photoswitchable fluorescent protein (Dendra2) was attached to its C-terminus. Before photoactivation, Dendra2 protein absorbs 488 nm light. After photoactivation by a 364 nm laser light, Dendra2 absorbs 543 nm light. In other words, pohotoactivation converts Dendra2 from a green to a red fluorescent protein. The mt-SOD1^G93A^-Dendra fusion protein was used to access mitochondrial dynamics and morphology. A plasmid, mt-SOD1-Dendra was also constructed as a negative control. Each of these plasmids was transfected into the FDB muscle of normal mice. Seven days after transfection, FDB muscle fibers expressing mt-SOD1-Dendra or mt-SOD1^G93A^-Dendra were isolated and incubated with TMRE. Figure **4A** shows that both mt-SOD1-Dendra and mt-SOD1^G93A^-Dendra fusion proteins were preferentially expressed in mitochondria. Remarkably, 90% (n=73) of muscle fibers expressing mt-SOD1^G93A^-Dendra showed aggregated fluorescent protein inside mitochondria. In contrast, not a single fiber expressing mt-SOD1-Dendra had aggregated fluorescent protein (n=36). In addition, 63% of fibers expressing mt-SOD1^G93A^-Dendra showed fragmented mitochondria compared to only 11% of fibers expressing mt-SOD1-Dendra (Figure **4D**). The time-dependent migration of Dendra fluorescent proteins was monitored using the method described earlier. Representative images are shown in Figure **5 A and B**. When examining the migration of mt-SOD1^G93A^-Dendra, fiber regions with aggregates and fragmented mitochondria were avoided. On average, mt-SOD1-Dendra migrated 8.8±1.1 m-steps in 18 min (n=20) while mt-SOD1^G93A^-Dendra migrated just 3.5±0.6 m-steps in the same period (n=16, P<0.01). The migration rates were determined by linear regression fits (Figure **5D**) and reported in Table **1**. We also examined local fiber regions with mt-SOD1^G93A^-Dendra aggregates and found that there was no significant movement of the fluorescent protein during the 20 min recording after the photoactivation, suggesting mitochondrial dynamics in those regions are more severely impaired. Overall, our data show that muscle fibers in normal mice expressing mutant mt-SOD1^G93A^-Dendra have significantly reduced mitochondrial dynamics compared to control fibers expressing mt-SOD1-Dendra. 

### Abnormal mitochondrial dynamics is associated with mitochondrial inner membrane depolarization

Figure **4A and D** show that mutant SOD1^G93A^ forms aggregates inside mitochondria and leads to fragmented mitochondrial network. As shown in Figure **4B**, some mitochondria with mt-SOD1^G93A^-Dendra aggregates (**a1** and **a2**) show reduced TMRE staining (**a1**.**1** and **a2.2**), which is indicative of mitochondrial inner membrane depolarization. The correlation between depolarization and aggregated fluorescent protein is also evident in Figure **4C**. We tested if mitochondrial depolarization alone (without SOD1^G93A^ mutation) altered mitochondrial dynamics. We found that incubation of normal muscle fibers with 200 nM FCCP (20 min) partially depolarized the inner membrane potential evoking a reduction of TMRE fluorescence intensity of about 19% (data not shown). To evaluate mitochondrial dynamics, the same FCCP challenge was applied to fibers expressing wild type SOD1 fusion protein (mt-SOD1-Dendra). After 20-min FCCP incubation, a small fiber region was photoactivated and the migration of red fluorescent protein from this region was measured. Figure **5C** shows that inner membrane depolarization significantly slowed the movement of the red fluorescent protein. The movement reduced from 8.8±1.1 m-steps (control) in 18 min to 4.7±0.9 m-steps following FCCP incubation (n=15, P<0.05). 

### Mutant SOD1^G93A^ promotes mitochondrial fission events by enhancing the function of mitochondrial fission protein Drp1

**Figure 6:**
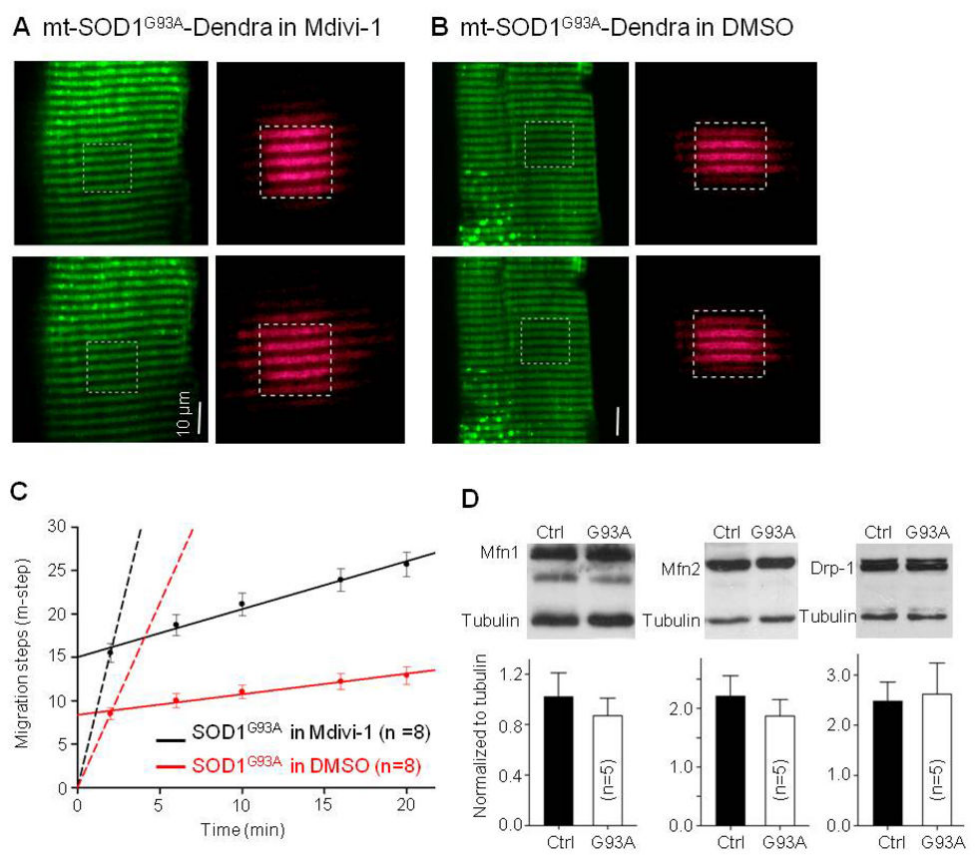
A-C. **The effect of Mdivi-1 on migration of mt-SOD1^G93A^-Dendra expressed in normal muscle fibers**. (**A**) Migration in the presence of Mdivi-1; (**B**). Migration in DMSO as a control. (**C**) The migration time course of mt-SOD1^G93A^-Dendra. Solid lines are the linear regression of the data after 2 minutes of the photoactivation. Dashed lines are the linear regression of the data in the first 2 minutes. The migration rates are listed in Table **1**. (P<0.01). Note, Mdivi-1 closely restored the migration rate of mutant mt-SOD1^G93A^-Dendra. (**D**) Western blot analysis of Mfn1, Mfn2 and Drp1 in skeletal muscle of G93A and control (ctrl) mice. There are no significant changes in expression level of these mitochondrial fusion/fission proteins in G93A muscle at the age of 2 month. (n=5 mice for control and G93A respectively).

Normal mitochondrial dynamics rely on the balance between fusion and fission processes. Reduced migration rate of SOD1G93A-Dendra fusion protein implies a net decrease in fusion events or a net increase in fission events. Mitochondrial fusion and fission events are regulated by key proteins such as Mitofusin (Mfn 1 and Mfn2) and dynamin-related protein 1 (Drp1) respectively (reviewed in [23]). We examined whether inhibition of mitochondrial fission protein Drp1 could restore normal mitochondrial dynamics in fibers expressing mutant SOD1^G93A^. Two groups of normal mice (3 in each) were transfected with mt-SOD1^G93A^-Dendra at the site of FDB muscle. In the next 7 days, one group received intraperitoneal injection of Mdivi-1, a specific inhibitor of Drp1 as described in Method section. The control group received the intraperitoneal injection of same amount of DMSO (vehicle). After the 7-day period, the FDB muscles were removed and mitochondrial dynamics were determined. Representative images are shown in Figure **6A & B**. In the Mdivi-1 group, mt-SOD1^G93A^-Dendra migrated 10.2±1.4 m-steps in 18 min (n=8). In the control group, mt-SOD1^G93A^-Dendra migrated just 4.4±1.0 m-steps in the same period (n=8, P<0.01). The migration rates were determined by linear regression fits (Figure **6C**) and reported in Table **1**. In addition, only 25% (n=35) muscle fibers in the Mdivi-1 group showed fragmented mitochondria comparing to 60% (n=42) of fibers in the control group. Thus, inhibition of mitochondrial fission by Mdivi-1 closely restored the mitochondrial network and the normal migration rate of the red fluorescent protein in muscle fibers expressing mutant SOD1^G93A^. 

We also conducted Western blot analysis to determine the expression level of key proteins involved in mitochondrial fusion and fission processes. Blots were prepared using skeletal muscle of young G93A mice at the same disease stage, a stage known to have altered mitochondrial dynamics (n=5 G93A mice and 5 age-matched control mice). Figure **6D** shows that there were a little reduction in the expression of mitochondrial fusion proteins Mfn 1 and Mfn2 and a little increase in mitochondrial fission protein Drp1 in G93A muscle. However these changes were not significant. The implication is that SOD1^G93A^ mutation does not significantly alter the protein expression level of these key proteins, but may enhance the function of Drp1 to promote mitochondrial fission events.

## Discussion

In this study, we discovered that skeletal muscle of an ALS mouse model G93A has abnormal mitochondrial dynamics at the age of two months when there is no significant axonal withdrawal and disease symptoms. We further demonstrated that similar abnormality of mitochondrial dynamics can be directly induced by overexpressing mutant SOD1^G93A^ protein in skeletal muscle of normal mice. Our data suggest that SOD1^G93A^ mutation drives ALS-associated muscle pathology in the absence of motor neuron degeneration.

Mitochondrial dynamics involves mitochondrial fission and fusion processes [22]. In skeletal muscle mitochondria form continuous networks in both transversal and longitudinal directions [32,33]. These networks are likely remodeled continuously by fission and fusion processes allowing contents to be exchanged between mitochondria. However, unlike other well studied cell types, skeletal muscle has very strict structural arrangement, the existence and relevance of mitochondrial dynamics is less understood in skeletal muscle [27]. Using mitochondrial targeted mt-PAGFP, we found that this fluorescent protein migrates through the mitochondrial network at two different rates. The migration is fast in the first 2 min following the photoactivation (Figure **2**, Table **1**). This initial fast migration is likely due to PAGFP diffusion between connected adjacent mitochondria. Later, further migration from the photoactivation site is much slower and likely depends on mitochondrial fission and fusion dynamics. Compared to the control muscle, the migration rate of mt-PAGFP in the G93A muscle was dramatically reduced in both time frames. This indicates that mitochondrial networks in G93A muscle are less extensive and undergo fewer fission and fusion events. 

Abnormal mitochondrial dynamics observed in G93A skeletal muscle could be 1) a consequence of the motor neuron degeneration or 2) directly caused by expression of the mutant ALS-causing protein SOD1^G93A^ in skeletal muscle. At two months of age G93A mice are asymptomatic and have no significant axonal withdrawal, but have altered mitochondrial dynamics in skeletal muscle. This implies that altered mitochondrial dynamics may be not due to axonal withdrawal. To verify this, we overexpressed SOD1^G93A^ in skeletal muscle of normal mice and reported that mutant SOD1^G93A^ targets to mitochondria and directly affects mitochondrial dynamics in skeletal muscle in the absence of motor neuron degeneration. 

SOD1 is a cytosolic enzyme that is also associated with different intracellular organelles including mitochondria [34]. Studies of ALS transgenic models have shown that accumulation of mutant SOD1 proteins inside mitochondria of motor neurons likely contributes to neuronal dysfunction [30,35]. ALS-causing mutant SOD1 was found selectively associated with mitochondria in central nervous system [36,37], but not in the liver [38]. Here, we tested for the first time if mutant SOD1 accumulates inside mitochondria of skeletal muscle. To monitor intracellular SOD1 trafficking in live skeletal muscle cells, we constructed SOD1-GFP and SOD1^G93A^-GFP plasmids and overexpressed these fusion fluorescent proteins in skeletal muscle of normal mice. Our results are the first evidence that both wild type SOD1 and mutant SOD1^G93A^ accumulate inside muscle mitochondria (Figure **3**). 

ALS is an age-dependent disease. It is likely that mutant SOD1^G93A^ causes mitochondrial toxicity in a time-dependent manner. Short time expression of mutant SOD1^G93A^ in the cytosol may not be enough to cause morphological and functional defects in skeletal muscle mitochondria. Indeed, we observed no differences in mitochondrial morphology between fibers expressing wild type SOD1-GFP or mutant SOD1^G93A^-GFP after 7 days of transfection (Figure **3**). To bypass the time-dependent accumulation process, we used mitochondria-targeted SOD1 fusion proteins. Mitochondrion-targeted expression resulted in marked morphological differences in mitochondria in muscle fibers expressing wild type SOD1 or mutant SOD1^G93A^. Seven days after transfection the majority of fibers expressing mt-SOD1^G93A^-Dendra had visible aggregated fluorescent protein inside mitochondria and fragmented mitochondria. In contrast, not a single fiber expressing wild type mt-SOD1-Dendra displayed this phenotype (Figure **4**). Protein aggregates and fragmented mitochondria have been reported in cultured motor neurons that were transfected with mitochondria-targeted mutant SOD1 [25,26,39]. Our similar results in muscle demonstrate that mitochondria in skeletal muscle are also directly subject to pathological actions of the ALS-causing mutant SOD1. 

Expression of wild type SOD1 (mt-SOD1-Dendra) inside mitochondria did not alter normal mitochondrial dynamics (see Figure **5** and Table **1**). The migration rate (after 2 min) of mt-SOD1-Dendra in normal muscle fibers (0.46 m-step/min) was almost the same as that of mt-PAGFP in normal muscle fibers (0.47 m-step/min). However, the expression of mutant mt-SOD1^G93A^-Dendra inside mitochondrial resulted in a 2.4-fold reduction in the migration rate in normal muscle fibers. This is similar to that observed in G93A muscle (3.6-fold reduction). These results provide strong evidence that accumulation of mutant SOD1^G93A^ inside mitochondria is able to disrupt the homeostasis of mitochondrial fission and fusion dynamics in skeletal muscle. Further, these results show that the disruption occurs in the absence of motor neuron degeneration. The migration rate (during the first 2 min) in fibers expressing mt-SOD1^G93A^-Dendra was reduced 1.6-fold, which is identical to that observed in the G93A muscle (Table **1**). This indicates that accumulation of mutant SOD1^G93A^ inside mitochondria also leads to discontinuity in mitochondrial network. Overall, these results suggest that normal muscle fibers expressing mutant mt-SOD1^G93A^-Dendra protein (not the wild type mt-SOD1-Dendra) reasonably reproduce the phonotype of G93A muscle (Figure **5**, Table **1**). 

G93A mouse model has systematic overexpression of mutant SOD1^G93A^ protein [2]. It is possible that the SOD1 mutation also causes impaired mitochondrial dynamics in motor neurons before onset of ALS. To our best knowledge, there are no reports on mitochondrial dynamics in live motor neurons derived from adult ALS mice with SOD1 mutations, because it is difficult to isolate live motor neurons from the spinal cord of adult mice to apply advanced gene transfection and live cell imaging methods. However, SOD1 mutation reduced mitochondrial dynamics in cultured motor neuron cell lines and primary motor neuron cultures derived from rodent embryos [25,26]. Here, by examining mitochondrial dynamics in skeletal muscle we provide new evidence supporting that impaired mitochondrial dynamics is likely a common pathologic defect caused by ALS-linked SOD1 mutations in both muscle and motor neuron during the disease progression. 

Normal mitochondrial dynamics rely on the balance between fusion and fission processes. Accumulating evidence has shown that abnormal mitochondrial fission mediated by Drp1 induces excessive mitochondrial fragmentation. This is a common pathway that leads to abnormal mitochondrial function critical to neuronal cell death [40,41]. We applied Mdivi-1, a specific inhibitor of mitochondrial fission protein Drp1 [29], to normal mice whose skeletal muscle was transfected with mutant SOD1^G93A^ (mt-SOD1^G93A^-dendra). Remarkably, inhibition of Drp1 restored mitochondrial network and the migration rate of the fluorescent protein. The results indicate that mutant SOD1^G93A^ promotes Drp1-based mitochondrial fission in skeletal muscle. We also examined expression levels of Drp1 and the mitochondrial fusion promoters (Mfn1/2) (Reviewed in [22,23]) in skeletal muscle of G93A mice at the age showing reduced mitochondrial dynamics. Expression of Mfn1/2 slightly decreased and Drp1 expression slightly increased (Figure **6D**). However, these changes were not statistically significant. It is likely that mutant SOD1^G93A^ does not significantly alter the expression level of these key proteins. In addition, it is also possible that changes in protein expression levels determined by Western blot may not be as sensitive as changes of mitochondrial dynamics detected in living muscle fibers. Also, Mfn1/2 and Drp1 function can be regulated in cells [24] independent of their expression level. Indeed, our Mdivi-1 experiments indicate that mutant SOD1^G93A^ promotes mitochondrial fission by enhancing the function of Drp1. In addition, skeletal muscle mitochondrial dynamics may involve different types of dynamic events other than fission and fusion, as recently discovered in cardiac muscle [42]. Thus, the mutant SOD1^G93A^ may also affect other unknown molecules that are involved in those muscle specific dynamic events [33], which may help explain the partial recovery of the migration by Mdivi-1 at the time period after 2 min of photoactivation. 

Abnormal mitochondrial dynamics in G93A muscle may be associated with mitochondrial membrane depolarization. Indeed, we found that partially depolarization of mitochondria by FCCP slowed the content exchange between mitochondria in normal muscle fibers. Localized mitochondrial depolarization was also evident in muscle fibers expressing mt-SOD1^G93A^-Dendra (Figure **4**) at the fiber region with protein aggregates. In young G93A muscle, mutant SOD1^G93A^ may only cause partial depolarization of mitochondria, which is not possible to be quantitatively evaluated by the non-ratio metric dye TMRE. However, the partial depolarization could disturb fission and/or fusion processes, explaining the abnormal mitochondrial dynamics in skeletal muscle of G93A mice before onset of other ALS disease symptoms. In our previous studies, we found that a portion of G93A muscle fibers have completely depolarized mitochondria in the fiber region near the neuromuscular junction (NMJ) [19,20]. Remarkably, we found here that all G93A muscle fibers tested had defective mitochondrial dynamics regardless of whether or not fibers had completely depolarized mitochondrial near their NMJ. This suggests that altered mitochondrial dynamics is a ubiquitous event that occurs early during ALS progression and precedes the localized NMJ defect. It is possible that accumulation of mutant SOD1^G93A^ inside mitochondria initially promotes partial depolarization of mitochondrial inner membrane potential and this enhances Drp1 function, which promotes the fission process. As a result, G93A muscle has less connected mitochondrial network and less exchange of mitochondrial contents. As observed in our previous study [19], mitochondria near NMJ are subject to elevated local Ca^2+^ activity. Mitochondria with defective dynamics may be more susceptible to Ca^2+^ overload at the site of NMJ and thus those mitochondria near NMJ are first to be completely depolarized. A subset of G93A muscle fibers with depolarized mitochondria near NMJ may be fibers that are further along in the disease process. 

In summary, this study is the first to show that mitochondrial dynamics is disrupted in G93A skeletal muscle. This dysfunction is associated with accumulation of mutant SOD1^G93A^ inside mitochondria and can occur independent of motor neuron degeneration. Our data provide direct evidence that mitochondria in skeletal muscle are primary points of pathological failure caused by an ALS-causing mutation SOD1^G93A^. The altered mitochondrial dynamics in G93A muscle likely contributes to overall disease progression. Future studies will be required to define the causal link between the defective muscle mitochondrial dynamics and the NMJ defect in ALS muscle as well as identify specific downstream pathways responsible for the defective mitochondrial dynamics and mitochondrial degeneration in ALS muscle. Importantly, this study suggests that development of agents that target and restore the homeostasis of mitochondrial dynamics could have therapeutic value in alleviating the muscle dysfunction associated with SOD1 mutation-related ALS.
